# Depression of intracranial self-stimulation in male and female rats by intraperitoneal lactic acid: effects of morphine, ketoprofen, and interactions with G-protein biased kappa opioid agonists

**DOI:** 10.1007/s00213-025-06800-3

**Published:** 2025-05-06

**Authors:** Thomas J. Martin, Conner W. Martin, Kevin J. Frankowski, Bruce E. Blough, Jeffrey Aubé, Laura M. Bohn, Sara R. Jones

**Affiliations:** 1 Pain Mechanisms Lab, Department of Anesthesiology, Wake Forest University School of Medicine, Winston-Salem, NC 27157 USA; 2 UNC Eshelman School of Pharmacy, University of North Carolina at Chapel Hill, Chapel Hill, NC USA; 3 Center for Drug Discovery, RTI International, Research Triangle Park, NC USA; 4 Departments of Molecular Medicine and Neuroscience, The Herbert Wertheim UF Scripps Institute for Biomedical Innovation & Technology, Jupiter, FL USA; 5 Department of Translational Neuroscience, Wake Forest University School of Medicine, Winston-Salem, NC USA

**Keywords:** Abdominal pain, Reinforcement, Kappa opioid, Operant behavior, Non-steriodal anti-inflammatory, Morphine

## Abstract

**Introduction:**

Numerous pharmacological classes of compounds have been explored as novel and efficacious alternatives to standard mu opioid agonist analgesics. We and others have described G-protein biased kappa opioid agonists as having potential utility as analgesics due to a lower propensity to produce sedation and dysphoria, which are thought to be mediated in large part through beta-arrestin signaling.

**Methods:**

Here we compare two G-protein biased kappa agonists that differ in their basic chemical scaffold, triazole 1.1 (Tr1.1) and isoquinolinone 2.1 (Iso2.1), for alteration of intracranial self-stimulation (ICSS) in male and female rats. Lactic acid (LA) was given i.p. at a concentration sufficient to produce moderate to severe depression of ICSS.

**Results:**

Neither Tr1.1 nor Iso2.2 reversed the effects of lactic acid at concentrations that produced significant depression of ICSS in either sex. Neither drug altered ICSS in the absence of lactic acid administration. In both males and females, morphine reversed the effects of i.p. lactic acid on ICSS and co-administration of Tr1.1 did not alter the dose–effect curve for morphine in either sex. Similar effects were observed for ketoprofen. Ketoprofen also reversed the effects of i.p. lactic acid on ICSS in both sexes in a dose-dependent manner, and co-administration of neither Tr1.1 nor Iso2.1 altered the ketoprofen dose–effect curve.

**Conclusions:**

These data suggest that these G-protein biased kappa agonists may lack sufficient efficacy or potency to alter the effects of opioids or NSAIDs against moderate to severe antinociceptive stimuli in rats, and development of more potent or efficacious compounds may be required to demonstrate efficacy in rat models of moderate to severe nociception.

## Introduction

Moderate to severe pain is currently treated primarily using mu opioid agonists and non-steroidal anti-inflammatory agents, both of which present significant adverse effects at therapeutic doses. For opioids, these adverse effects include respiratory depression, sedation, constipation, and significant abuse liability. Kappa opioid agonists act as analgesics, however their sedative and dysphoric effects have precluded their clinical development (French and van Rijn 2022). In the last several years, identification of kappa opioid agonists that display signaling bias for G-protein- relative to beta-arrestin-activated second messengers has offered promise of analgesics that are devoid of the unwanted side effects of prototypical kappa opioid agonists (Liu-Chen and Huang 2022). Novel kappa opioid agonists have been developed that stimulate G-protein mediated pathways with greater potency or efficacy than beta-arrestin mediated pathways, and it is postulated that G-proteins are involved in the analgesic properties while beta-arrestin signaling is involved in the sedating and dysphoric effects of kappa opioids (Ehrich et al. 2015; Ho et al. 2018; Kaski et al. 2021; Lovell et al. 2015). The extent of the bias towards one signaling pathway over the other by these compounds is determined in cell-based assays however, and the relative potency and efficacy for producing antinociceptive versus sedation or dysphoria in vivo must be determined empirically. We have previously published that a G-protein biased triazole-based kappa opioid agonist displays antinociceptive properties against a mild abdominal inflammatory pain stimulus without evidence of sedation or dysphoria (Brust et al. 2016b). This compound, termed triazole 1.1 (Tr1.1) also does not demonstrate abuse potential using intracranial self-stimulation in rats as the measure of abuse liability (Brust et al. 2016b). In agreement, we also have demonstrated that Tr1.1 does not depress dopamine release in nucleus accumbens slices, unlike the typical kappa agonist U50,488H, an effect that is postulated to be related to dysphoria, although others suggest inhibition of dopaminergic neurons is not required for the aversive properties of typical kappa opioids in rodents (Brust et al. 2016b; Ehrich et al. 2015). Other studies with Tr1.1 have shown that this compound decreases the abuse liability of oxycodone, while enhancing the antinociceptive effects, suggesting that Tr1.1 has the potential of being used as an adjuvant with mu opioid agonists for treatment of pain (Zamarripa et al. 2021).

In this study, we sought to compare the effects of Tr1.1 and a different G-protein biased kappa agonist based on an isoquinolinone scaffold, Iso2.1, in both male and female rats (Zhou et al. 2013a). We used intraperitoneal injection of lactic acid as a nociceptive stimulus, which has been studied extensively using several different behavioral endpoints and candidate analgesic compounds. Inflammation is thought to be a key component of nociception induced by this method since non-steroidal anti-inflammatory drugs consistently reverse or inhibit the behavioral effects of lactic acid injection (Altarifi et al. 2022; Baldwin et al. 2022; Diester et al. 2021; LaCourse et al. 2025; Santos et al. 2024). Generally kappa opioid agonists are more potent or more efficacious in producing antinociception in male compared to female rodents, however these effects are dependent upon the strain of rat or mouse and the pain stimulus applied (Rasakham and Liu-Chen 2011). Females have also been shown to be less sensitive to the dysphoric effects of kappa agonists (Russell et al. 2014). While our previous studies demonstrated efficacy against a relatively mild pain stimulus, the most pressing unmet clinical need is for treatment of moderate to severe pain, for which mu opioid agonists are the primary standard of care. We therefore sought to determine if either Tr1.1 or Iso2.1 demonstrated efficacy against a moderate pain stimulus, if Tr1.1 demonstrated evidence of potentiation of a mu opioid agonist at non-sedating or non-dysphoric doses, and if the analgesic effects of either Tr1.1 or Iso2.1 could be potentiated by a non-steroidal anti-inflammatory agent, in either male or female rats.

## Methods

### Subjects

Subjects consisted of 19 male and 17 female Fisher 344 rats (Enivgo Inc., Raleigh, NC) that were 7–10 weeks of age at the beginning of the experiments. Of these animals, 16 males and 16 females were successfully trained to perform ICSS within the criteria given below. Male and female animals were housed in separate rooms adjacent to the laboratory containing the behavioral equipment and maintained on a reversed 12:12 h light:dark schedule (dark 05:00–17:00). Animals were given ad lib access to standard rat chow (ProLab RM500, LabDiet, Arden Hills, NM) and water except during experimental sessions. All housing and experimental rooms are within an AAALAC accredited facility. All procedures were performed in accordance with the Guide for the Care and Use of Laboratory Animals as adopted by the National Institutes of Health, met the guidelines approved by the International Association for the Study of Pain, and were approved by the Wake Forest University School of Medicine Institutional Animal Care and Use Committee.

### Drugs and chemicals

The G-protein biased kappa agonists triazole 1.1 (Tr1.1) and isoquinolinone 2.1 (Iso2.1) were synthesized and prepared as previously published (Brust et al. 2016b; Morgenweck et al. 2015; Slauson et al. 2015; Zhou et al. 2013b). These compounds were dissolved in DMSO initially, then diluted in a 1:1 ratio with Tween-80 to achieve the final concentration of 12 mg/ml. Morphine sulfate was obtained from the Drug Supply Program of the National Institute on Drug Abuse. Ketoprofen was obtained from Zoetis Inc. (Kalamazoo, MI) as a 100 mg/ml sterile solution and diluted in 0.9% (w/v) saline. DMSO and Tween-80 were obtained from Sigma-Aldrich Inc. (St. Louis, MO). Isoflurane and penicillin G procaine were obtained from Aspen Veterinary Resources (Liberty, MO). Ketamine was obtained from West-Ward (Eatontown, PA) and Xylazine was obtained from Bimeda Inc. (Le Sueur, MN). Sterile saline (0.9% w/v, pH 7.4) and lactated Ringers solutions were obtained from Baxter Healthcare Corp. (Deerfield, IL). All drugs and chemicals were authenticated by the vendors or by co-authors (KJF, GLM, BEB, JA) in the case of Tr1.1 and Iso2.1 using previously published methods cited above.

### ICSS electrode implantation

Electrodes for ICSS were implanted at the approximate rostrocaudal and dorsoventral center of the medial forebrain bundle. Rats were anesthetized using isoflurane induction (5% saturation) followed by ketamine (50 mg/kg, s.c.) and xylazine (10 mg/kg, s.c.). The scalp was shaved and prepped with povidone iodine (Medline Industries Inc., Northfield, IL) followed by 70% isopropanol. Rats were placed into a stereotaxic frame and a 1.5 cm incision was placed on the midline, the scalp retracted, and underlying fascia blunt dissected to expose the skull surface. A burr hole was placed through the skull at the appropriate coordinates (−2.5 mm from bregma, 1.7 mm lateral from midline for both sexes) and the electrode (2 channel twisted pair platinum, MS303/6-B, P1 Technologies, Roanoke, VA) was affixed to the skull (depth from skull surface, male 8.5 mm; female 7.7 mm) using cranioplastic cement and self-tapping stainless steel screws (Amuza Inc., San Diego, CA). The scalp incision was closed using an interrupted mattress suture pattern with 4–0 Vicryl (Ethicon LLC, Guaynabo, PR). Animals were administered 75,000 U of penicillin G procaine s.c. diluted in 3 ml of lactated Ringers solution and given 3 mg/kg s.c. of ketoprofen. Animals were kept on a thermal blanket in a clean polycarbonate cage until recovery of normal movement.

### ICSS training and drug administration

#### Apparatus

All components unless otherwise noted were purchased from Med Associates Inc. (St. Albans, VT). All sessions were conducted in standard rat operant chambers (ENV-008-VP) containing two levers, two stimulus lamps located above each lever, a food hopper located between each lever, a tone generator, and a counterbalance arm with a two channel commutator (PHY-015–2). ICSS stimulation was provided through a dual programmable ICSS stimulator (PHM-152/2). Operant chambers were enclosed within a sound- and light-attenuating chamber containing a ventilation fan and a light mounted to one upper corner.

#### Procedure

The procedure for training and maintenance of ICSS has been published previously (Ewan and Martin 2011). After a minimum of two weeks of recovery from electrode implantation surgery, rats were trained to press a lever for square wave cathodal-anodal stimulation. The electrode was connected to the commutator through a stainless steel mesh encased cable (P1 Technologies). The initial frequency used to engender lever presses was 156 Hz and the current amplitude was adjusted from 50 to 300 µA to optimize response rate without evidence of motor impairment by visual observation. The initial starting point for determining the optimal current amplitude was 80% of the motor threshold. Each stimulation was 0.5 s in duration and signaled by operation of the tone. The stimulus light above the active lever was turned on to indicate ICSS availability. Each lever press resulted in the stimulus light being turned off and delivery of 0.5 s of stimulation accompanied by operation of the tone and illumination of the houselight, followed by all lights and the tone being turned off and a time-out period of 0.5 s with lever presses producing no programmed consequences. Once the optimal current amplitude was estimated, a current response curve was obtained by varying the current amplitude across 1.5 orders of magnitude in 10 discreet trials, with the frequency set to 156 Hz for all trials. The current amplitudes used were evenly spaced in incremental log units with the estimate of the optimal amplitude comprising the mid-point in the array. Each trial began with non-contingent delivery of five 0.5 s stimulations at the amplitude available for that trial each separated by a 0.5 s time-out. The stimulus light above the active lever was turned on and lever presses were reinforced by delivery of 0.5 s of stimulation. Each trial was 1 min in duration and the lowest available amplitude was used for the first trial, followed by the other amplitudes in successive trials in ascending order. The session consisted of six 10 min components, with each component comprised of 10 separate trials. Once the current intensity response curves were stable, the optimal current amplitude used for subsequent sessions was the one that produced 85% of the maximal response rate, individually determined for each animal. After determination of the optimal current amplitude, frequency response curves were obtained. The structure of the trials and components were similar, with the exception that frequency was varied across trials within each component according to the array (Hz): 156, 136, 118, 103, 90, 78, 68, 59, 52, 45. Sessions consisted of 3 components of 10 trials each, followed by a time-out period for drug treatment, followed by an additional 3 components of 10 trials each. Sessions were conducted on weekdays only during the dark phase of the light:dark cycle.

#### Drug and lactic acid treatment

The protocol for drug and lactic acid treatment was similar to that published previously (Brust et al. 2016b). Once frequency–response curves were stable, defined as 5 successive sessions by which the EF50 and Rmax (see below) did not vary by more than 15% from the mean, rats were administered s.c. Tr1.1, Iso2.1, morphine, or ketoprofen, or their respective vehicles (1:1 DMSO:Tween-80 for Tr1.1, Iso2.1; saline for morphine, ketoprofen) in a volume of 1 ml/kg immediately after conclusion of the third component of the ICSS session. Animals were then returned to the operant chamber and 30 min later were administered i.p. either lactic acid or saline in a volume of 1 ml/kg. Animals were then returned to the operant chamber within 2 min for the remaining 3 components of the session. Order of drug administration was randomized according to a Latin square design as published previously (Brust et al. 2016b).

#### Data analysis and statistics

All data were analyzed using GraphPad Prism 9.0 (GraphPad Software, San Diego, CA). Frequency rate curves were generated for each animal and each session by averaging response rate at each frequency across the second and third components of the session prior to drug or vehicle treatment, and across all 3 components following drug or vehicle and i.p. lactic acid or saline administration. The frequency yielding half maximal response rate (EF50) and maximal response rate (Rmax) were determined from the averaged frequency rate curves prior to and after drug/vehicle and saline/lactic acid treatment using iterative curve fitting to the dose–effect curve function with variable Hill slope. Only data from curve fits with r^2^ of 0.8 or greater were included in subsequent analyses. The logEF50 and logRmax values were used for subsequent analyses as these values are normally distributed rather than the non-logarithmic values. Group data were tested for normal distribution and equal variances, and the Geissner-Greenhouse correction was applied when variances were different between groups. The differences in logEF50 or logRmax were determined by subtracting the values obtained from the curves after drug/vehicle and lactic acid/saline treatment from those obtained prior to treatment. A positive change in EF50 indicates a shift to the right following treatment (higher frequencies necessary to attain comparable response rates, i.e. less reinforcing), while a negative change in Rmax indicates a downward shift after treatment (maximal response rates are lower after treatment, i.e. less reinforcing). Data for the change in logEF50 and change in logRmax were the dependent measures with drug dose or vehicle and i.p. lactic acid or saline as the independent variables. These data were analyzed using two-way ANOVA, and post-hoc analyses were performed using Dunnett’s t-test with drug vehicle serving as control. While not all subjects received all treatments, there was substantial overlap in the treatments for individual subjects. We performed 4 separate ANOVAs to determine the interactions between morphine and Tr1.1, and between ketoprofen and Tr1.1 or Iso2.1. For morphine, we performed two separate two-way ANOVAs with morphine dose and Tr1.1/vehicle as the independent variables, one in the presence of i.p. lactic acid and the other in the presence of i.p. saline. For ketoprofen, we also performed two separate two-way ANOVAs with ketoprofen dose and either Tr1.1/vehicle or Iso2.1/vehicle as the independent variables, both with i.p. lactic acid injection. We performed two additional one-way ANOVAs to determine the effects of Tr1.1 or Iso2.1 alone compared to vehicle following administration of either lactic acid or vehicle. Post-hoc comparisons were made using Dunnett’s t-test with drug vehicle serving as the control. As we performed 6 separate ANOVAs with significant overlap in the subjects used, the p-value for significance was adjusted to 0.05/6 or 0.0083. For comparisons of baseline ICSS measures and effects of LA only between male and female rats, student’s t-test was used with a p-value of 0.05 for significance. Data from male and female rats were analyzed separately since the lactic acid concentrations used were different between male and female rats as described in results below.

## Results

### Baseline ICSS comparisons between male and female rats

**Fig. 1:**
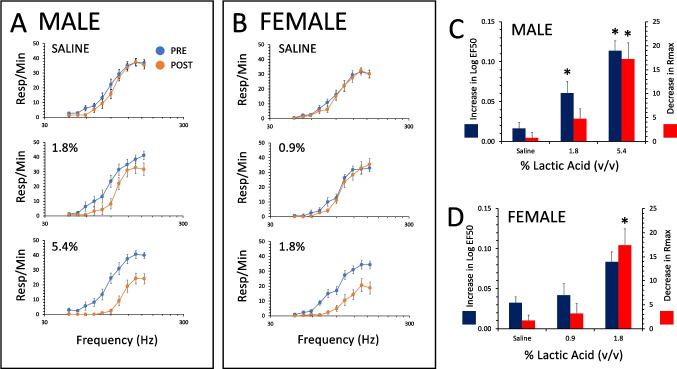
Lactic acid depression of ICSS in male and female rats. Shown are rate-frequency curves and changes in either LogEF50 or Rmax (mean ± SEM). Stimulation of the VTA maintained similar rates of responding across all frequencies between male (A, N = 16) and female (B, N = 12) rats, and administration of saline i.p. had no effect on ICSS in either sex. Lactic acid depressed ICSS in a concentration dependent manner in males (A,C, N = 11–16) and females (B,D, N = 8–12). In males, 1.8% lactic acid i.p. increased the EF50 but had no effect on the Rmax, while 5.4% increased the EF50 and decreased the Rmax (C). In females 0.9% lactic acid i.p. had no effect on ICSS, while 1.8% lactic acid i.p. decreased the Rmax (D) to a similar extent as 5.4% in males. *, significantly different from saline i.p

The frequency–response curves were not different between male and female rats at baseline with respect to either the log EF50 (t = 0.16, p = 0.9) or the Rmax (t = 1.1, p = 0.3) (Fig. 1). Response rate across all frequencies was frequency-dependent [F(9,234) = 123, p < 0.0001] and did not differ between males and females [F(1,26) = 2.8, p = 0.1] (Fig. 1A,B). The intensities used across subjects also did not differ between males (163.1 ± 11.4 µA, mean ± SEM) and females (201.7 ± 19.8 µA) (t = 1.8, p = 0.09).

### Effects of i.p. lactic acid on ICSS in male and female rats

In male rats, i.p. lactic acid increased the EF50 and decreased the Rmax in a concentration-dependent manner (Fig. 1A,C). The increase in EF50 was dependent on lactic acid concentration [F(2,38) = 20.1, p < 0.0001] with the increase after both 1.8% and 5.4% being significantly different from saline. The decrease in Rmax following i.p. lactic acid was likewise concentration dependent [F(2,38) = 12.9, p < 0.0001], with i.p. administration of 5.4% significantly decreasing Rmax compared to saline.

In female rats, preliminary studies (N = 4) using 5.4% lactic acid eliminated responding for ICSS almost entirely and the animals did not return to baseline within 2 weeks after a single administration of this concentration i.p. (not shown). This concentration was not used for any subsequent studies in females. The LogEF50 was not increased significantly by any concentration of lactic acid [F(2,27) = 0.2, p = 1.0] but did however decrease the Rmax in a concentration-dependent manner [F(2,27) = 8.9, p = 0.001] (Fig. 1B,D). The Rmax was decreased significantly following administration of 1.8% LA compared to saline or 0.9%, with no difference between the effect of 0.9% from saline administration (Fig. 1B,D). The extent to which i.p. administration of 5.4% LA decreased the Rmax in males was not significantly different from the effect of 1.8% LA administration on the Rmax in females (t = 0.5, p = 0.6). Therefore, the effects of subsequent drug treatments and combinations were assessed in males using 5.4% LA and in females using 1.8% LA to provide levels of noxious input that were relatively equivalent regarding the effect on ICSS for males and females for determination of drug efficacy.

### Effects of Tr1.1 and Iso2.1 on LA depression of ICSS in male and female rats

**Fig. 2:**
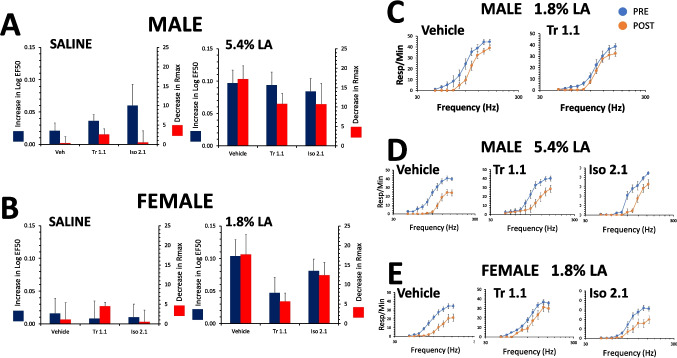
Effects of Tr1.1 and Iso2.1 on ICSS in male and female rats. Shown are rate-frequency curves and change in LogEF50 or Rmax (mean ± SEM) following administration of vehicle, Tr1.1 or Iso2.1 in combination with i.p. administration of either saline to determine potential sedative/dysphoric effects or with LA to determine potential antinociceptive effects. (**A)** The change in LogEF50 and Rmax following pretreatment s.c. with vehicle, Tr1.1 or Iso2.1 with administration of saline or 5.4% LA i.p. is shown in male rats calculated from the rate-frequency curves shown in D. (**B)** The change in LogEF50 and Rmax following pretreatment s.c. with vehicle, Tr1.1 or Iso2.1 with administration of saline or 1.8% LA i.p. is shown in female rats calculated from the rate-frequency curves shown in E. N = 8 to 12/group. **(C)** Male rats were administered either vehicle or Tr1.1 (24 mg/kg, s.c.) 30 min prior to administration of 1.8% LA. PRE refers to rate-frequency curves determined prior to drug and lactic administration and POST refers to the average of 3 rate-frequency curves after administration. (**D)** Vehicle, Tr 1.1 or Iso2.1 were administered to male rats prior to administration of 5.4% LA i.p. and rate-frequency curves determined as in C. (**E)** Vehicle, Tr1.1 or Iso2.1. were administered to female rats prior to administration of 1.8% LA i.p. and rate-frequency curves determined as in C and D. *, significantly different compared to vehicle administration

To assess possible dysphoric or sedative effects of the biased agonists, the maximum feasible dose of each kappa agonist based on solubility was administered to rats given saline i.p. Neither Tr1.1 (24 mg/kg, s.c.), Iso2.1 (24 mg/kg, s.c.) nor vehicle significantly affected ICSS in either male or female rats (Fig. 2A,B). Neither the ΔEF50 or ΔRmax were significantly different from zero for vehicle, Tr1.1, or Iso2.1 (p > 0.05). Additionally, the effect of vehicle, Tr1.1 or Iso2.1 did not differ significantly for either sex when compared using ANOVA for either the increase in LogEF50 [males: F(2,33) = 2.4, p = 0.3; females: F(2,28) = 0.3, p = 0.7] or decrease in Rmax [males: F(2,33) = 0.9, p = 0.4; females F(2,28) = 0.6, p = 0.6]. In males given 1.8% LA i.p., Tr1.1 (24 mg/kg, s.c.) pretreatment prevented the increase in LogEF50 (t = 2.2, p = 0.048) following i.p. administration of 1.8% LA (0.003 ± 0.02) compared to s.c. vehicle administration (0.06 ± 0.02), but did not reverse the effect on the decrease in Rmax (Tr1.1: 5.2 ± 1.8, Veh: 3.8 ± 1.8; t = 0.5, p = 0.6), similarly to our previous reports with this compound (Fig. 2C). When the noxious stimulus was increased to 5.4% LA i.p. in males, neither Tr1.1 (24 mg/kg, s.c.) nor Iso2.1 (24 mg/kg, s.c.) significantly reversed the effect on either the LogEF50 [F(2,36) = 0.09, p = 0.9] or the Rmax [F(2,36) = 1.1, p = 0.4] (Fig. 2A,D). In females, neither Tr1.1 nor Iso2.1 produced a significant reversal of the effect of 1.8% LA on the LogEF50 compared to vehicle [F(2,28) = 1.7, p = 0.2] or the Rmax [F(2,28) = 3.4, p = 0.05] (Fig. 2B,E).

### Effects of morphine and ketoprofen on LA depression of ICSS in male and female rats and interactions with Tr1.1 and Iso2.1

#### Interactions between Tr1.1 and morphine

**Fig. 3:**
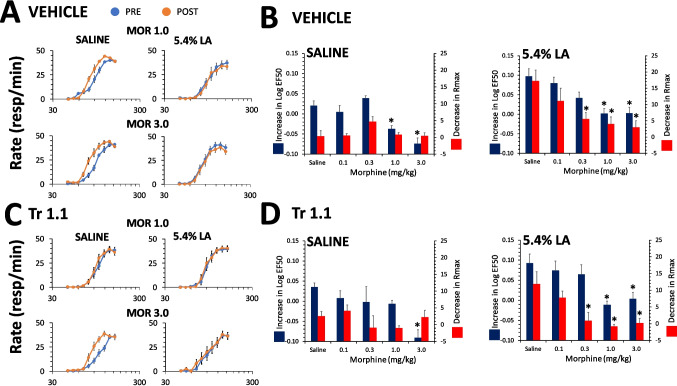
Effects of morphine alone and in combination with Tr1.1 on ICSS in male rats. Shown are rate-frequency curves and change in LogEF50 or Rmax (mean ± SEM) following administration of morphine (0.1–3.0 mg/kg, s.c.) in the presence or absence of Tr1.1 (24 mg/kg, s.c.), with saline or 5.4% lactic acid i.p. (**A)** Male rats were administered 1 or 3 mg/kg of morphine and vehicle 30 min prior to administration of saline or 5.4% LA i.p. PRE refers to rate-frequency curves determined prior to drug and lactic administration and POST refers to the average of 3 rate-frequency curves after administration. (**B)** Increase in LogEF50 or decrease in Rmax (mean ± SEM) are shown for morphine dose–effect curves when co-administered with vehicle 30 min prior to administration of saline or 5.4% LA i.p. *, significantly different from saline co-administered with vehicle. (**C)** Male rats were administered 1 or 3 mg/kg of morphine and 24 mg/kg of Tr1.1 30 min prior to administration of saline or 5.4% LA i.p. Pre and Post reference the same conditions as in A. (**D)** Increase in LogEF50 or decrease in Rmax (mean ± SEM) are shown for morphine dose–effect curves when co-administered with 24 mg/kg Tr1.1 30 min prior to administration of saline or 5.4% LA i.p. *, significantly different from saline co-administered with 24 mg/kg Tr1.1. N = 8 to 12/group for each graph

In males, in the absence of noxious input morphine facilitated ICSS as reported previously, decreasing the EF50 compared to saline in a dose-responsive manner [F(4,102) = 15.8, p < 0.0001] while having no effect on the Rmax [F(4,102) = 1.1, p = 0.4] (Fig. 3A,B). There was no significant effect of co-administration of Tr1.1 compared to vehicle on the morphine dose–effect curve for either the shift in the LogEF50 [F(1,102) = 0.04, p = 0.8] or the Rmax [F(1,102) = 0.2, p = 0.6] and no interaction between Tr1.1 co-administration and morphine dose [LogEF50: F(4,102) = 1.5, p = 0.2; Rmax: F(4,102) = 2.3, p = 0.07] (Fig. 3C,D, left panels). Using Dunnett’s post-hoc test, the LogEF50 was decreased following administration of 1.0 or 3.0 mg/kg of morphine with co-administration of vehicle, but only following administration of 3.0 mg/kg of morphine with co-administration of Tr1.1 (Fig. 3C,D, left panels). In the presence of noxious input, morphine produced a dose-dependent reversal of the increase in the LogEF50 with 5.4% LA administration [F(4,111) = 11.8, p < 0.0001], however Tr1.1 co-administration did not alter the effect of morphine [F(1,111) = 0.01, p = 0.9] and there was no interaction between Tr1.1 co-administration and morphine dose [F(4,111) = 0.2, p = 0.9] (Fig. 3B,D right panels). Following administration of 5.4% LA, the ΔLogEF50 was significantly different from saline with pretreatment of 1.0 and 3.0 mg/kg of morphine in the presence and absence of Tr1.1 co-administration (Dunnett’s) (Fig. 3B,D right panels). Morphine likewise produced a dose-dependent reversal of the effect of 5.4% LA on the Rmax [F(4,111) = 9.6, p < 0.0001], and this effect was not significantly increased by Tr1.1 co-administration [F(1,111) = 5.4, p = 0.02] with no interaction between Tr1.1 co-administration and morphine dose [F(4,111) = 0.07, p = 1.0] (Fig. 3B,D right panels, red bars).

**Fig. 4:**
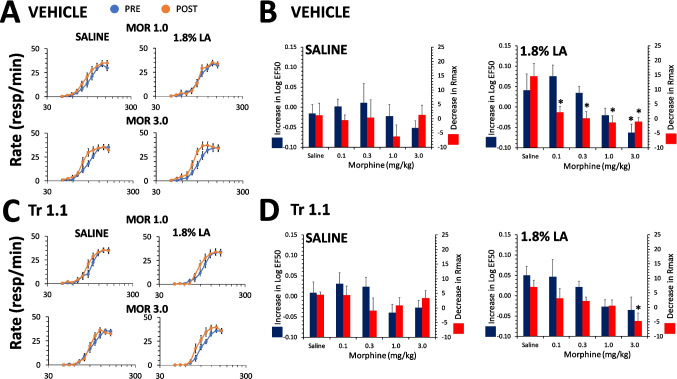
Effects of morphine alone and in combination with Tr1.1 on ICSS in female rats. Shown are rate-frequency curves and change in LogEF50 or Rmax (mean ± SEM) following administration of morphine (0.1–3.0 mg/kg, s.c.) in the presence or absence of Tr1.1 (24 mg/kg, s.c.), with saline or 1.8% lactic acid i.p. (**A)** Female rats were administered 1 or 3 mg/kg of morphine and vehicle 30 min prior to administration of saline or 1.8% LA i.p. PRE refers to rate-frequency curves determined prior to drug and lactic administration and POST refers to the average of 3 rate-frequency curves after administration. (**B)** Increase in LogEF50 or decrease in Rmax (mean ± SEM) are shown for morphine dose–effect curves when co-administered with vehicle 30 min prior to administration of saline or 1.8% LA i.p. *, significantly different from saline co-administered with vehicle. (**C)** Female rats were administered 1 or 3 mg/kg of morphine and 24 mg/kg of Tr1.1 30 min prior to administration of saline or 1.8% LA i.p. PRE and POST reference the same conditions as in A. (**D)** Increase in LogEF50 or decrease in Rmax (mean ± SEM) are shown for morphine dose–effect curves when co-administered with 24 mg/kg Tr1.1 30 min prior to administration of saline or 1.8% LA i.p. *, significantly different from saline co-administered with 24 mg/kg Tr1.1. N = 8 to 12/group for each graph

In the absence of noxious input in females, morphine did not produce a dose-dependent effect on either the LogEF50 [F(4,98) = 1.9, p = 0.1] or the Rmax [F(4,98) = 0.7, p = 0.6] (Fig. 4A,B). Tr1.1 co-administration did not alter the effect of morphine on either parameter (EF50: [F(1,98) = 0.7, p = 0.4]; Rmax [F(1,98) = 1.9, p = 0.2]) and there was no interaction with morphine dose (EF50: [F(4,98) = 0.2, p = 0.9]; Rmax: [F(4,98) = 0.3, p = 0.9] (Fig. 4C,D). In females morphine produced a dose-dependent reversal of the effects of 1.8% LA on ICSS (Fig. 4A,B). The increase in LogEF50 following administration of 1.8% LA was reduced by morphine in a dose-dependent manner [F(4,95) = 5.8, p = 0.0003]. There was no effect of Tr1.1 on the morphine dose–effect curve [F(1,95) = 0.02, p = 0.9] and no interaction between Tr1.1 treatment compared to vehicle and morphine dose [F(4,95) = 0.3, p = 0.9] (Fig. 4C,D). The ΔLogEF50 was different from saline pretreatment only at the highest dose of morphine tested (3.0 mg/kg) with vehicle co-administration and was not significantly different from saline at any dose of morphine co-administered with Tr1.1 with 1.8% LA administration. Similarly, morphine reversed the effects of 1.8% LA on depression of the Rmax in a dose-dependent manner [F(4,95) = 8.4, p < 0.0001], and Tr1.1 co-administration did not alter the effects of morphine [F(1,95) = 0.5, p = 0.5] and there was no interaction between Tr1.1 co-administration and morphine dose [F(4,95) = 1.3, p = 0.3] on the Rmax (Fig. 4 C,D). Morphine significantly reduced the effect of 1.8% LA on the Rmax at all doses with vehicle co-administration compared to saline, however only at the highest dose with Tr1.1 co-administration (Dunnett’s) (Fig. 4B,D). Therefore, morphine showed antinociception at doses in females that did not potentiate ICSS in the absence of noxious input, and Tr1.1 co-administration did not alter the antinociceptive effect of morphine.

#### Interactions between Tr1.1 or Iso2.1 and ketoprofen

**Fig. 5:**
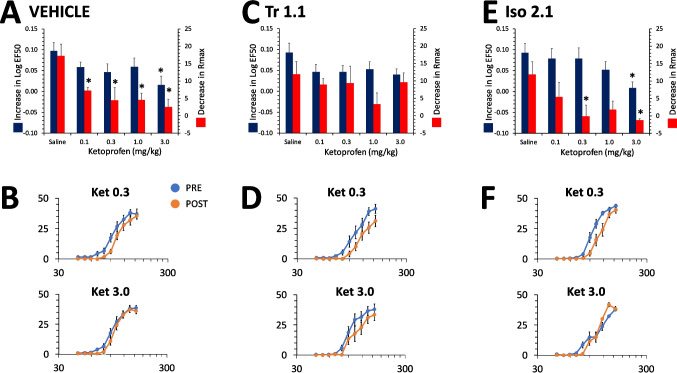
Effects of ketoprofen alone and in combination with Tr 1.1 and Iso 2.1 on the effects of 5.4% LA on ICSS in male rats. (**A)** Dose–effect curve for ketoprofen co-administered with vehicle s.c. 30 min prior to administration of 5.4% LA i.p. for increase in Log EF50 or decrease in Rmax (mean ± SEM). *, significantly different from saline co-administered with vehicle. (**B)** Male rats were administered 0.3 (top) or 3.0 mg/kg of ketoprofen (bottom) in combination with vehicle s.c. 30 min prior to administration of 5.4% LA i.p. PRE refers to rate-frequency curves determined prior to drug and lactic administration and POST refers to the average of 3 rate-frequency curves after administration. (**C)** Dose–effect curve for ketoprofen co-administered with 24 mg/kg of Tr1.1 s.c. 30 min prior to administration of 5.4% LA i.p. for increase in Log EF50 or decrease in Rmax (mean ± SEM). *, significantly different from saline co-administered with vehicle. **(D)** Male rats were administered 0.3 (top) or 3.0 mg/kg of ketoprofen (bottom) in combination with 24 mg/kg of Tr1.1 s.c. 30 min prior to administration of 5.4% LA i.p. (**E)** Dose–effect curve for ketoprofen co-administered with 24 mg/kg of Iso2.1 s.c. 30 min prior to administration of 5.4% LA i.p. for increase in Log EF50 or decrease in Rmax (mean ± SEM). *, significantly different from saline co-administered with vehicle. (**F)** Male rats were administered 0.3 (top) or 3.0 mg/kg of ketoprofen (bottom) in combination with 24 mg/kg of Iso2.1 s.c. 30 min prior to administration of 5.4% LA i.p. N = 8 to 12/group for all graphs. * significantly different from saline

As with morphine and consistent with previous results, ketoprofen reversed the effects of LA administration on ICSS in male rats in a dose-dependent manner for both the LogEF50 [F(4,107) = 3.6, p = 0.008] and the Rmax [F(4,107) = 4.2, p = 0.003] (Fig. 5A,B). Compared to saline treatment, only the highest dose of ketoprofen (3 mg/kg) reversed the effect of 5.4% LA on the LogEF50, while all doses produced a significant reversal of the effect of 5.4% LA on the Rmax (Fig. 5A,B). Co-administration of Tr1.1 did not alter the effects of ketoprofen on either the LogEF50 [F(1,107) = 0.003, p = 0.96] or Rmax [F(1,107) = 0.5, p = 0.48], with no interaction between Tr1.1 co-administration and ketoprofen dose [LogEF50: F(4,107) = 0.2, p = 0.9; Rmax: F(4,107) = 1.4, p = 0.2] (Fig. 5C,D). In males, co-administration of Iso2.1 did not change the effects of ketoprofen on either the LogEF50 [F(1,103) = 0.3, p = 0.6] or the Rmax [F(1,103) = 3.2, p = 0.07] and there was no interaction with respect to ketoprofen dose [LogEF50: F(4,103) = 0.3, p = 0.8; Rmax: F(4,103) = 0.1, p = 1.0] (Fig. 5E,F). Since ketoprofen alone has not been found to produce any significant effects on ICSS in the absence of noxious input, the interaction between ketoprofen and either Tr1.1 or Iso2.1 was not assessed in the absence of 5.4% LA i.p. administration.

**Fig. 6:**
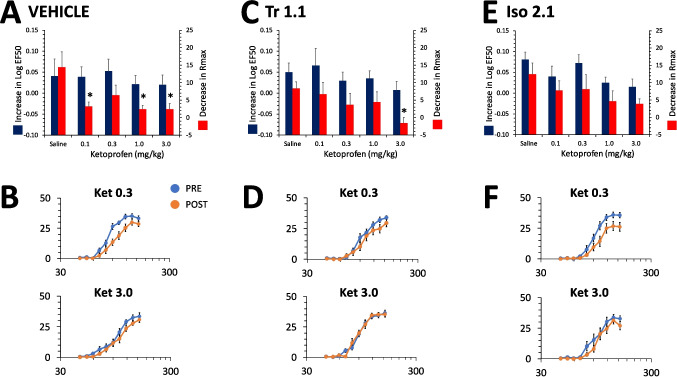
Effects of ketoprofen alone and in combination with Tr 1.1 and Iso 2.1 on the effects of 1.8% LA on ICSS in female rats. (**A)** Dose–effect curve for ketoprofen co-administered with vehicle s.c. 30 min prior to administration of 1.8% LA i.p. for increase in Log EF50 or decrease in Rmax (mean ± SEM). *, significantly different from saline co-administered with vehicle, p ≤ 0.05. (**B)** Female rats were administered 0.3 (top) or 3.0 mg/kg of ketoprofen (bottom) in combination with vehicle s.c. 30 min prior to administration of 1.8% LA i.p. PRE refers to rate-frequency curves determined prior to drug and lactic administration and POST refers to the average of 3 rate-frequency curves after administration. (**C)** Dose–effect curve for ketoprofen co-administered with 24 mg/kg of Tr1.1 s.c. 30 min prior to administration of 1.8% LA i.p. for increase in Log EF50 or decrease in Rmax (mean ± SEM). *, significantly different from saline co-administered with vehicle. **(D)** Female rats were administered 0.3 (top) or 3.0 mg/kg of ketoprofen (bottom) in combination with 24 mg/kg of Tr1.1 s.c. 30 min prior to administration of 1.8% LA i.p. (**E)** Dose–effect curve for ketoprofen co-administered with 24 mg/kg of Iso2.1 s.c. 30 min prior to administration of 1.8% LA i.p. for increase in Log EF50 or decrease in Rmax (mean ± SEM). *, significantly different from saline co-administered with vehicle. (**F)** Female rats were administered 0.3 (top) or 3.0 mg/kg of ketoprofen (bottom) in combination with 24 mg/kg of Iso2.1 s.c. 30 min prior to administration of 1.8% LA i.p. N = 8 to 12/group for all graphs. * significantly different from saline

In females the same range of doses of ketoprofen did not produce a significant effect in reversing the effect of 1.8% LA on the LogEF50 [F(4,88) = 0.6, p = 0.7] (Fig. 6A,B) and there was no effect of Tr1.1 co-administration on the ketoprofen dose–effect curve [F(1,88) = 0.03, p = 0.9] and no interaction between Tr1.1 co-administration compared to vehicle with respect to ketoprofen dose [F(4,88) = 0.3, p = 0.9] (Fig. 6C,D). There was a dose-dependent effect of ketoprofen on reversal of the effect of 1.8% LA on the Rmax [F(4,88) = 4.6, p = 0.002], with all doses producing a significant reversal of the effect compared to saline except 0.3 mg/kg (Fig. 6A,B). There was no effect of Tr1.1 co-administration on the dose–effect curve for reversal of the effects of 1.8% LA on the Rmax [F(1,88) = 0.7, p = 0.4] and no interaction between Tr1.1 versus vehicle co-administration and ketoprofen dose [F(4,88) = 1.1, p = 0.3] (Fig. 6C,D). In females, co-administration of Iso2.1 also did not alter the effects of ketoprofen on either the LogEF50 [F(1,88) = 0.5, p = 0.5] or the Rmax [F(1,88) = 0.7, p = 0.4] and there was no interaction with respect to ketoprofen dose [LogEF50: F(4,88) = 0.3, p = 0.9; Rmax: F(4,88) = 0.3, p = 0.9] (Fig. 6 E,F). The interaction between either Tr1.1 or Iso2.1 and ketoprofen was not examined in female rats in the absence of noxious input for the same rationale as noted above for male rats. Neither Tr1.1 nor Iso2.1 significantly increased the antinociceptive effects of ketoprofen in either male or female rats.

## Discussion

The present study builds upon previous findings that have shown the G-protein biased kappa agonist Tr1.1 produces antinociception in the absence of behavioral disruption in male rats using ICSS as the behavioral endpoint and 1.8% lactic acid i.p. as the nociceptive stimulus (Brust et al. 2016b). We used Fisher 344 rats in the present study to remain consistent with our previous publication. We further demonstrate that the antinociceptive effects of Tr1.1 depend on the strength of the nociceptive stimulus, in that it did not reverse the effects of 5.4% LA i.p. on ICSS in males. In females, Tr1.1 did not reverse the effects of 1.8% lactic acid on ICSS. Kappa opioid agonists have been shown to be more effective in females than males following oral surgery, however with the current experimental paradigm we did not find evidence of increased antinociception using the present compounds (Fillingim and Gear 2004). The other G-protein biased kappa agonist tested, Iso2.1, failed to provide any evidence for antinociception at doses up to 24 mg/kg in either males or females, the highest practical dose that could be tested due to solubility.

Several pertinent sex differences emerged from these data. The first noteworthy difference is in the sensitivity of male versus female rats to LA-induced depression of ICSS. A lower concentration of LA was required in females compared to males to produce a similar extent of depression in maximal response rate for ICSS (1.8% versus 5.4%), despite similar baseline LogEF50 and Rmax values in the same frequency range and current intensities required to obtain frequency-rate curves. Others have shown that ICSS reinforcement is similar in male and female rats, in agreement with the present study (Michaels et al. 2007; Stratmann and Craft 1997). These data suggest that female rats are more susceptible to behavioral depression in the presence of abdominal inflammation than males, however such a conclusion would require additional studies examining the extent of inflammation resulting from different doses of lactic acid in males and females, as well as using other relevant behavioral endpoints. Clinical data show that females present more often than males with abdominal inflammatory pain, and that this pain is often of greater severity (Kim and Kim 2018; Manson 2010). There were also differences in the effect of morphine on responding for ICSS between males and females in the absence of noxious input, with females failing to show potentiation of ICSS by these doses of morphine. The ability of morphine to facilitate ICSS in rats appears to be strain dependent however, and in Sprague–Dawley rats, females have demonstrated greater facilitation of ICSS than males with acute mu opioid agonists (for review see (Negus and Moerke 2019)). The present data in males agree with our previous findings in Fisher 344 male rats (Brust et al. 2016b; Ewan and Martin 2011). At face value, these data suggest that morphine only produces reversal of abdominal inflammatory pain at doses with significant abuse liability in males. In females however, morphine reversed the effects of abdominal inflammatory pain at doses that failed to potentiate ICSS, suggesting greater separation between reinforcement and antinociception relative to males. Further experiments examining other nociceptive stimuli and other behavioral measures are necessary to strengthen this conclusion, certainly. There is little evidence in the clinical literature to support such a conclusion, however, and the relative lack of sensitivity to morphine for facilitation of VTA ICSS in females may indicate differential effects of mu opioids on the dopaminergic component of reinforcement between males and females in the Fisher 344 strain compared to Sprague–Dawley rats that are more commonly-used in preclinical studies. As noted above, differences have been noted between Sprague–Dawley and Fisher 344 male rats regarding facilitation of VTA ICSS by mu opioid agonists. Facilitation of VTA ICSS likely occurs primarily through dopaminergic mechanisms, either directly or indirectly (review (Negus and Moerke 2019)). Several non-dopaminergic mechanisms by which mu opioids produce reinforcement have been postulated, and while a review of this literature is beyond the scope of this discussion, this aspect merits further investigation, as well as the possible role of strain differences.

Tr1.1 reduces oxycodone self-administration in male rats when these two drugs are mixed (Zamarripa et al. 2021). Tr 1.1 also reduces reinforcing effects of oxycodone at doses that produce fewer unwanted effects in nonhuman primates (Huskinson et al. 2022; Zamarripa et al. 2024). These studies and the present data suggest that Tr1.1 co-administration with a mu-opioid agonist may increase the separation between antinociception and abuse liability, at least in males. In the present study, we did not find that Tr1.1 administered as a pretreatment altered the dose–effect curve for morphine’s effects on ICSS, either in the presence or absence of i.p. lactic acid. The differences in the present data compared to those cited above could be due to a number of factors including morphine vs. oxycodone, route of administration, strain or species, and the antinociceptive stimulus.

We have previously shown that ketoprofen inhibits the effects of i.p. LA on depression of ICSS in males (Brust et al. 2016a). We were interested to determine if there were positive interactions between ketoprofen and Tr1.1 or Iso2.1, which have similar G-protein bias profiles but distinct chemical scaffolds. Ketoprofen primarily reversed the effects of LA on decreasing the Rmax of the rate-frequency curves within the same dose range in male and female rats, and neither Tr1.1 nor Iso2.1 substantially increased these effects of ketoprofen. The use of NSAIDS is common for inflammatory pain states, however significant unwanted effects are dose-limiting clinically. It does not appear from these data that either G-protein biased agonist has the potential to either increase the efficacy of NSAIDs against abdominal inflammatory pain, nor provide dose-sparing effects against moderate to severe abdominal inflammatory pain on their own in either sex.

There are several noteworthy limitations to the present study. While the logarithmic values for EF50 and Rmax were found to be normally distributed, tests for normality have limited statistical power for sample sizes less than 30–50 subjects. While i.p. lactic acid is accepted as an inflammatory pain stimulus in rodents as cited above, we did not explicitly test for inflammatory mediators in the abdominal cavity, nor assess stimulation of primary sensory neurons in the abdomen per se. Rather these data are empirical in nature, using classical behavioral pharmacology and building upon the literature cited above. We also did not examine sex differences quantitatively, as the effects of 5.4% lactic acid given i.p. to females produced long-term adverse effects on behavior in females that could not be reconciled with the experimental design. The time points used for testing the G-protein biased kappa agonists were based on previous data showing brain penetrance and effects of Tr1.1 on kappa opioid G-protein activation in brain using Fisher 344 rats (Brust et al. 2016b). We used a similar time point for Iso2.1, however such pharmacokinetic studies on this compound have not been performed.

In conclusion, Tr1.1 shows efficacy against mild, but not moderate, abdominal inflammatory pain in rats. Further study is warranted to determine the clinical potential of a mu-opioid agonist together with a G-protein biased kappa agonist in mild to moderate pain states, particularly in males. The present data suggest that females are more sensitive to i.p. LA than males while being less sensitive to potentiation by morphine. Given the lack of effect of Tr1.1 found here, it is important to assess effects of G-protein biased kappa opioid agonists of greater efficacy or potency, given the solubility limits of the triazole compounds that precludes examining higher doses. Kappa agonists have been recently described that are both more potent and have similar or greater G-protein biased signaling as Tr 1.1, and these compounds would be interesting candidates for further pharmacological study (Trojniak et al. 2024).

## Data Availability

The data for this study will be made available from the corresponding author upon request.
